# Hotel Dieu

**Published:** 1832-11

**Authors:** 


### HOSPITAL REPORTS.

### HOTEL DIEU.

*Acute Nephritis, arising without obvious Cause, quickly followed by Death. Considerable Swelling of the inflamed Kidney, with Suppuration and Softening of its Substance; Wasting of the other Kidney.* Treated by Dr. Dance.

A mason, thirty-three years old, of a strong and hardy constitution, was admitted into the Hôtel Dieu on the 6th of June, having suffered for three weeks with a settled pain in the left lumbar region: the pain came on suddenly; preceded, however, according to the man's account, by œdema of the lower limbs, but of which no trace remained. When admitted, besides the pain in the loins, which was greatly increased on pressure, there was a peculiar swelling of the parts, and the whole abdominal parietes were so much distended as to prevent a satisfactory examination of the deeply-seated viscera; his countenance expressed the greatest anxiety; pulse frequent and small; the skin hot, but the tongue natural. He was ordered to be bled, and the blood exhibited a buffy coagulum.

On the following day, the anxiety continued undiminished; besides which, the tongue was red and dry, the lumbar pains extended to the epigastrium, and he passed his urine frequently, in very small quantities. It may be remarked, that neither nausea nor vomiting were present; he had no pain nor retraction of the testicle, nor any swelling in the groin; symptoms the absence of which is mentioned because it was considered from the first to be a case of nephritis. He was bled largely twice during the day; the coagula were firm and buffed each time.

On the third day he had shiverings, increasing to horripilation; the subcutaneous veins were scarcely perceptible, and he endeavoured to warm himself by rolling his bedclothes round him; his breathing was hurried and anxious; pulse small, frequent, and wiry; urine scanty, and excruciating pain in the lumbar region, especially if touched. He was bled a fourth time, and cataplasms ordered, with liquorice ptisan; and towards evening the heat and anxiety diminished.

On the fourth day, the shivering returned in the morning; the anxiety was extreme; the tongue foul and dry. In the evening a remission occurred: the febrile symptoms lessened, but the pain continued in the loin and epigastrium; the form of which *was apparently increased* in size.

On the fifth day, the fever returned early in the morning, but without being preceded by shivering; respiration laborious; speech scarcely audible; pulse hard and frequent; urine very high coloured, almost black, and depositing a slight whitish sediment. He was bled a fifth time, and the blood was buffy as before.

On the sixth day, the symptoms were very discordant; for while the skin was cool and the pulse small, without being preternaturally quick, the countenance was haggard, the respiration irregular, anxious, and suffocating; the patient seeming to labour to relieve his chest from the oppression of its contents, by violent expirations. The pain in the loin and epigastrium continued as severe as ever. A warm bath was ordered, but the patient found himself worse for its use, and his respiration became still more embarrassed.

On the seventh day, the symptoms continued the same, with the exception of the sensation of coldness, now extending to the whole surface of the body, and being increased to such a degree as to make it unpleasant for any one to touch him. His countenance exhibited signs of intense suffering; his head was bent forcibly backwards; jactitation incessant; convulsions and stiffness of the limbs; intellect oppressed; pulse irregular, thready, and only about seventy per minute.

The rapid progress of this disease, its daily exacerbations preceded by shiverings, and the impotency of antiphlogistic remedies, persuaded the trial of an injection of sulphate of quinia, and the administration of the same medicine by the mouth, which were accordingly prescribed: but, an hour after the visit, his pulse became insensible, his respiration more feeble, a cadaverous chill spread over the body, and death prevented the administration of the last-ordered medicine.

*Autopsy*. Great rigidity of the body, no emaciation. ***Head***: Infiltration of fluid beneath the arachnoid, two or three spoonsful of serum in the ventricles. *Thorax*: Lungs distended with a frothy fluid, especially towards their posterior edges; no other unhealthy appearance; heart in a natural state. *Abdomen*: Mucous membrane of the stomach slate-coloured; peyerian glands distinct, but no ulceration of the bowels. *Urinary system*: Left kidney four times the natural size, forming a solid heavy tumor, extending from the concavity of the diaphragm to the crest of the ileum. A little whitish fluid pus was collected at the top of the kidney beneath its proper tunic, which was easily removed from the subjacent structure; the cortical portions were of a livid brownish red. Numerous abscesses, from the size of a pea to that of a filbert, were dispersed throughout the whole of the parenchymatous substance, but the chief were towards the circumference, and contained a whitish inodorous pus, partly infiltrated. Besides this, the substance of the kidney was softened, so that it resembled a half-hepatised lung; its colour a reddish brown, marked with white streaks and spots of suppuration. These signs of intense inflammation were chiefly noticeable in the cortical substance. The pelvis was of an ordinary size; it contained neither calculi nor any purulent matter; and its walls were healthy. The left ureter was in a natural state. The right kidney had wasted away; it consisted of a firm tissue, but was not larger than a hen's egg, and exhibited various lobulations and irregularities on its surface, such as are found in the fœtal state. Pelvis and ureter on this side healthy. The bladder was much contracted, and formed within numerous saccules, half filled with muddy urine, in which were noticed some muciform flakes.

This inflammation of the kidney, which might well be called parenchymatous, and at the same time phlegmonous, differed from ordinary nephritis in this, that it was the proper substance of the viscus that was affected, and not the mucous membrane alone, which lines the excretory ducts. It was then pre-eminently a case of nephritis, in which the symptoms were not present which mark the catarrhal form of this disease. In short, excepting the local pain corresponding with the seat of the organ affected, and of which the intensity was in proportion to the degree of the inflammation, the other symptoms were not the ordinary ones of nephritis, and might have led to the momentary belief of the existence of a virulent intermittent fever. All the mischief however arose from the kidney, which was found excessively disorganised; its substance softened, infiltrated with pus, and studded with abscesses; and its volume greatly increased.

It is worthy of observation, that this violent nephritis occurred without any apparent cause, and was not the effect of calculi or other foreign bodies developed in the parenchymatous structure of the kidney, as is frequently the case. Perhaps the atrophy noticed in the left kidney, by compelling its fellow to perform the functions of both, favored the development of this fatal inflammation. However this might be, the progress of the disorder was so violent and rapid, that the most energetic antiphlogistic treatment (five copious bloodlettings,) was proved to be wholly inefficient.

---

### *Cases and Observations on certain Forms of Gangrene,* by M. Dupuytren.

### *Arteritis: Coagulation of the Blood; Symptomatic Gangrene; Death.*

A woman, named Sigolet, forty years of age, and of regular habits, was admitted into the Hôtel Dieu, July 15, 1832, for incipient gangrene of the right leg. She was of delicate constitution, but had always enjoyed good health, with the exception of having recently suffered from cramp in the right lower extremity. She had dull pain, of but little severity, which was first experienced in the right iliac region; from this it descended along the inner part of the thigh, and then to the back part of the leg, till it reached the sole of the foot and toes. The parts were affected with prickings and acute shooting, which passed into fixed burning pain. It was only then, being about eight or ten days before her admission into the hospital, that the foot began to grow cold: purple spots appeared, and the pain became so great as to prevent sleep.

On his visit of the 16th, M. Dupuytren found that the foot and leg of the right side were swollen to twice their natural size as far as the knee: the skin was tense and shining, as in phlegmonous erysipelas. The foot was of a deep purple towards the toes, which became less intense a little farther up, and shewed itself in marbled patches on the leg. The cuticle was removed at some points. The part was extremely cold at the upper third of the leg, and this continued increasing down to the toes: the sensibility was diminished in the direct ratio of the heat: the power of motion still remained entire, a circumstance easily understood, when it is considered that most of the muscles of the foot are situated on the leg, and chiefly at its upper third. The pulsation of the femoral arteries of the left side was felt to be full and regular, while in the right one the pulsations were so extremely weak as to be almost imperceptible: the artery seemed to be converted throughout its whole extent into a hard and nearly incompressible cord. The diagnosis made by M. Dupuytren was "arteritis, of which the gangrene is but a symptom." The patient was ordered to lose three paletts of blood, to have a large poultice applied to the limb, and slop diet.

The bleeding had the effect of alleviating the pain and inducing sleep. It was repeated next day, and on the 18th its effects were still more marked: the pain was nearly gone, there was less swelling, and both the heat and sensibility had returned at several points. On the other hand, the mortified part was covered with vesicles containing dark fluid, which bursting, discovered the cutis, black, gangrenous, and fœtid. The limb was enveloped in camphorated spirits.

On the 22d she was bled for the third time. For some days after, the gangrene appeared to be arrested about four fingers' breadth below the knee: it was supposed that the whole thickness of the limb beneath was mortified. The movements of the foot were now entirely lost, but the leg could be bent on the thigh. Nevertheless, whether the nerves had resisted the sloughing, or whether it was from a phenomenon analogous to that which takes place after amputation, the patient still occasionally experienced exquisite pain in the foot. Up to this time the treatment had not overcome the diseased process, but yet it had appeared to be retarded. However, about the end of July, notwithstanding two more bleedings, the icy coldness pervaded the knee, and progressively extended upwards. By the 11th of August the mortification implicated the lower part of the knee-pan, the coldness being well marked two inches higher up, and no pulsation was now perceptible in any part of the femoral artery. On the 16th the lower third of the thigh was involved in the gangrene, and then the strength, which had hitherto held out, began to break very rapidly: diarrhœa came on, and she died on the 19th, being thirty-five days after her admission.

*Autopsy.* The body and limb externally presented nothing to remark in addition to what is above described as present during life. The vessels were first examined in the part of the thigh which had remained sound, at least which was not gangrenous. About the middle of the limb the artery, though natural in appearance, was diminished in size, and contained a thread-like coagulum, of a red colour, and which was supposed to have been formed after death.

Towards the crural arch the vessel regained its ordinary size: it was hard, incompressible, and filled with a clot, red on the surface, and slightly adherent to the sides of the artery; within it was of greyish colour, and appeared to be formed of fibrine. This was continued up as far as the origin of the primary iliac, and even extended a little into the left iliac, but without obliterating it. The internal iliac was also obstructed by a clot of the same kind. The right crural vein was filled with a firm red clot. The vessels of the left lower extremity, the aorta, and the heart, were all nearly empty. Between the parts which remained sound and the gangrene was interposed a space of from two to three inches, which had been cold during life: there the cellular texture displayed the appearance of a reddish grey and very marked capillary injection, alternated: lower down, at the margin of the gangrene, the vascularity disappeared. The skin was black, hard, and dry as parchment; the subjacent cellular tissue of a yellowish grey; the aponeuroses pale; the muscles of a bright red, moist, and streaked with layers of cellular tissue more white than natural; the nerves rose-coloured; the vessels in the ham containing a greyish clot, like that found higher up; the bones of a pale grey, with the periosteum firmly adherent.

M. Dupuytren remarked, that this constituted an excellent example of what used to be called "spontaneous mortification," the "gangrene of old persons," "gangrene without a known cause;" but that these appellations must now be discarded, as pathology has explained the disease. Gangrene of the extremities may take place from very different causes. In old persons ossification is generally found in the arteries of the limb, and then too the dead parts are almost always dry, and not tumefied, as in the preceding case. In other instances the evil is caused by an organic affection of the heart, a circumstance remarked by Corvisart. In adults, at an earlier period, we also sometimes meet with arterial ossification; or gangrene may result in them from ligature of the great vessels, events sufficiently well known. But a more frequent cause, and to the merit of first discovering which M. Dupuytren lays claim, is acute inflammation of the artery, with coagulation of the blood, obliteration of the vessel, and consequent complete interruption of the circulation. Formerly, reasoning from views purely theoretical, and likening this kind of mortification to what was seen from frost-bite, it was asserted that it occurred most commonly in winter; whereas just the reverse is true, for it is particularly in the summer months that it occurs. The woman whose case is above detailed was attacked during the period of the greatest heat. M. Dupuytren it was who first pointed out this fact, and it was the circumstance of its greater frequency in summer which led him to suspect that there was some error in the old hypothesis. The above case is also curious, as exhibiting the disease in the female, (women being decidedly less liable to it than men, and at an early period of life (forty). It is remarkable, however, that M. Trioen has seen two cases in young girls, one of twenty-three and the other of only nine years. At first the disease is purely local; the respiration and circulation, as well as the functions of the brain and of the alimentary canal, remain undisturbed; and it is only when the disease has made some progress, doubtless only when absorption has commenced from the putrid mass, that the general system sympathises, and fatal consequences ensue.

The progress of the gangrene is marked by an extraordinary sense of cold and paleness of the part. It is not, as might be supposed, a reduction of temperature analogous to what takes place in the dead body, and which only happens because the corpse assumes the temperature of the surrounding medium; it is an icy coldness, and is greater than that of the dead body, nay, the thermometer sinks lower than in the air, or even than in a current of water. M. Dupuytren long since made many experiments on this point, and ascertained that the temperature of the part about to fall into this kind of gangrene became less than that of the dead body. The pulsations in the artery, as seen above, cease, or nearly so, and in the situation of the vessel a firm cord is felt. It was predicted before death that the artery would be found obstructed as high as the aorta; but in one respect the diagnosis was not complete; for though the artery was obstructed at the aorta, yet lower down the clot was a mere thread, and it was the coagulum in the vein which gave the feeling of a cord along the inner side of the thigh. During fifteen years M. Dupuytren witnessed the exclusive use of bark and similar means, which proved quite unavailing: since the cause of the disease has been better ascertained, and repeated bleeding adopted, "we have cured two thirds, and even three fourths, of our patients."

[The reporter asks why opium, as recommended by Pott, was not used in conjunction with the bleeding; and why, as the disease still advanced, amputation was not had recourse to, in the manner practised by Larrey? a practice which has been followed by some well-marked examples of success.]

*Ascites: Gangrene of the Foot supervening immediately after Tapping.*

A woman, named Bourbon, aged fifty-six, apparently exhausted by fatigue and privation, so that she looked twenty years older, laboured under dropsy. About three months ago, the ascites having become very considerable, she had the operation of tapping performed. Immediately after, the foot swelled, and in two days (to use her own expression) it was "dead." The gangrene had reached the ankle, and the bones had separated at the articulation, and the soft parts a little higher up, the tibula and fibula projecting, and being in a state of necrosis. The patient had no pain; the arteries pulsated freely, and no ossification could be detected. The tapping required to be repeated, and was followed by no inconvenience. The belly being thus emptied, led to the detection of two hard and painful tumors within it, one to the right, the other on the left of the umbilicus; but they exhibited no pulsation, and the circulation was not disturbed.

Here was a case in which the cause of the gangrene remained unknown. M. Dupuytren expected to have found some lesion of the abdominal circulation to explain it, but was compelled to abandon this conjecture.

### *Peculiar Mortification of the Cellular Membrane.*

M. Dupuytren afterwards made some remarks upon another form of gangrene, hitherto undescribed. We sometimes see slow inflammations, with little heat or pain, attack individuals of different age, sex, and constitution. It is a subcutaneous swelling, which takes at least two months to be developed, and which sometimes requires twelve months for this purpose. At last the skin becomes purple, fluctuation is felt, and, on opening it, scrofulous pus is evacuated, which discovers a slough at the bottom of the abscess. The wound is dressed in the usual way, but no progress is made, and the slough does not separate. Two or three months pass before it is detached, and then it comes away in one piece, white, hard, and excessively fœtid, and appearing to be constituted entirely of cellular membrane; and yet, what is extraordinary, not becoming softened by putrefaction. After this, the bottom of the wound is seen to be very unequal, partly red and partly white, and it is still a long time ere healthy granulations make their appearance. Several cases of this kind have lately been treated in the Hôtel Dieu: in one, admitted on the 8th of August last, a very large slough of the above description separated from a chronic tumor on the right side of the thorax.

